# Multicentric giant cell tumor around the knee

**DOI:** 10.4103/0019-5413.32048

**Published:** 2007

**Authors:** Anil Salgia, SK Biswas, Rahul Agrawal, Vishavdeep Goyal

**Affiliations:** Department of Orthopaedics, Padmashree Dr. D. Y. Patil Medical College, Hospital and Research Centre, Pimpri, Pune - 411 018, Maharashatra, India

**Keywords:** Amputation, arthrodesis, arthroplasty, curettage, giant cell tumor

## Abstract

A case of multicentric giant cell tumor with synchronous occurrence in all three bones around the knee is reported here in view of its rarity. A 33-year-old average built male reported with complaints of severe pain, gradually increasing swelling around the right knee. A 3 × 2 cm swelling was present on the lateral aspect of the distal end of the right femur and a 3 × 3 cm swelling on the proximal part of the right tibia. Plain X-ray of right knee showed subarticular eccentrically located expansile lytic lesion in the lateral tibia condyle, lateral condyle of femur and patella. Fine needle aspiration cytology and subsequent histology ascertained the diagnosis of giant cell tumor of the bone. The patient was treated successfully with curettage, bone grafting and methyl methacrylate cementing (Sandwich technique).

Giant cell tumor is commonly seen in young adults 20-40 years of age with slight female predominance and contributes 5% of total primary neoplasms.^1^ The most common sites are the distal femur, proximal tibia and distal radius. Giant cell tumors are solitary lesions but rarely 1-2% may be metachronously multicentric. The synchronous occurrence is further rare. Hence a case of multicentric giant cell tumor of the bone with synchronous occurrence around the knee is reported.

## CASE REPORT

A 33-year-old average built male reported with complaints of severe pain, gradually increasing swelling around the right knee and inability to bear weight on the right lower limb for last one month following history of trivial trauma. Pain was continuous, increased in night and was not relieved by rest. Patient had history of continuous and dull aching pain in the lower limb, used to get relieved by analgesics, for the last one and a half years. There was no history of fever, vomiting, weight loss, hemoptysis or loss of appetite. No other bony swelling was present elsewhere in the body.

On examination a swelling of 3 × 2 cm was present on the lateral aspect of the distal end of the right femur and a 3×3 cm swelling on the proximal part of the right tibia. The skin over the swelling was stretched but mobile. Local tenderness was present with rise of temperature. There were no subcutaneous dilated veins. Movements of the right lower limb were not possible due to pain. Hemogram and blood counts were within normal limits. Acid and alkaline phosphatase and serum calcium were within normal range.

**Figure 1:**
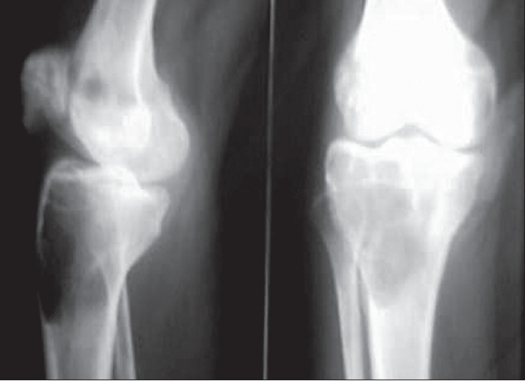
Pre Operative X-ray lateral and A.P. view shows Eccentric lytic lesions in upper end tibia, lower end femur & patella

**Figure 2:**
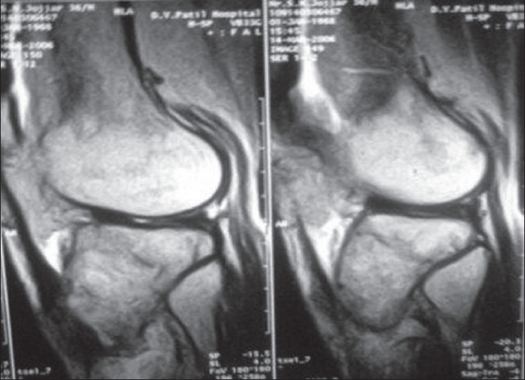
MRI (sagittal section) shows sub articular eccentrically located lesions in the lateral tibial plateau, lateral femoral condyle & patella

Plain X-ray of right knee showed subarticular eccentrically located expansile lytic lesion in the lateral tibia condyle, lateral condyle of femur and patella [Figure 1]. The MRI showed subarticular eccentrically located lesion in the lateral tibial condyle with serpiginous area of altered marrow signal intensity of the lower end of femur and patella to suggest the possibility of bone infarct and likely to resemble a multifocal giant cell tumor. The fine needle aspiration cytology done showed giant cell lesion of bone [Figure 2].

**Figure 3:**
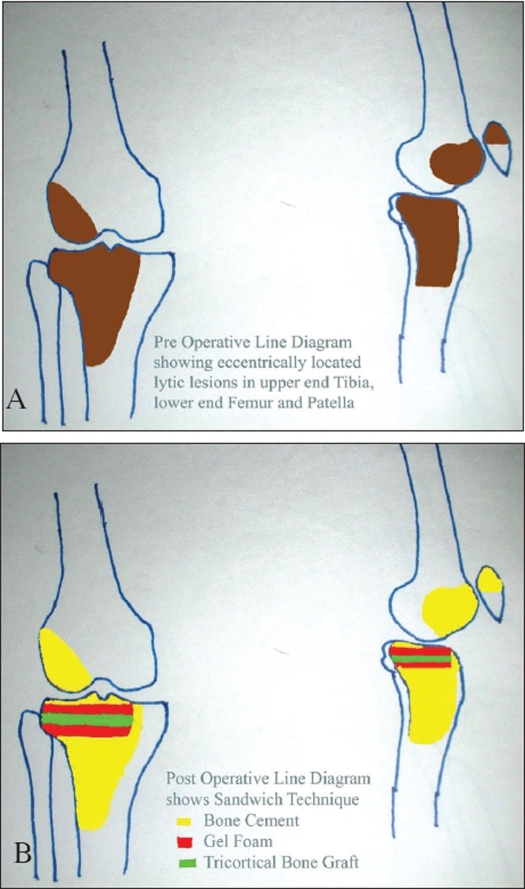
A) Line Digram showing eccentrically located lytic lesions in lower end femur, upper end tibia & patella, B) Line Digram showing Sandwich Technique

Through a lateral para-patellar incision, lesions were explored. Femoral articular surface was found intact. Tibial articular surface was minimally damaged on its lateral corner. Patellar articular surfaces were not damaged. All the lesions from the femur, tibia and patella were thoroughly curetted and were chemically and electrically cauterized with phenol and electric cautery. A tricortical graft (6×4 cm) was harvested from the left iliac crest. A block of gel foam 5 × 4 × 1 cm was placed underneath the tibial articular surface in the subchondral area and the harvested bone graft was tailored to be placed below the gel foam and the remaining bony gap was filled with bone cement (Sandwich Technique). Bone cement was also filled in the right femoral condyle and patellar lesion after curettage. After saline lavage the wound was closed [Figures 3A and B].

Postoperative period was uneventful. Non-weight-bearing physiotherapy in the form of knee movements, quadriceps and hamstring strengthening exercises were started the next day. Partial weight-bearing was allowed after seven days with hinged long leg knee brace. On the 12^th^ postoperative day sutures were removed, patient was ambulated with full weight-bearing after three weeks.

On first follow-up after one and a half month, patient showed excellent recovery in the form of quadriceps and hamstring strength with right knee range of movements from 5° to 110°. Follow up X-ray shows maintenance of joint space and no recurrence of lesions. Patient was able to bear full weight on the operated limb. On subsequent follow-up after six months onwards, patient had full range of painless movements of right knee with no clinical or radiological evidence of recurrence of lesion. Patient has resumed his job of driving vehicles and is doing all his daily activities without any difficulty.

**Figure 4:**
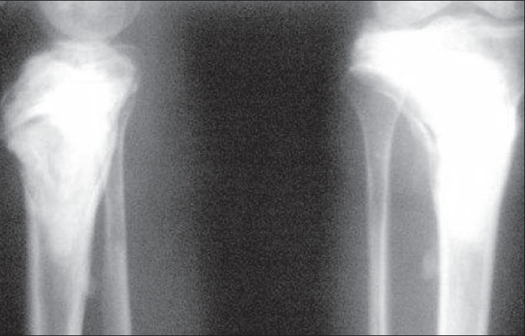
X-ray (lateral and AP view) after 1 year shows no evidence of recurrence

Patient was reviewed clinically and radiologically one year after surgery and there is no evidence of recurrence of the lesions in any of the bones. Patient has started his routine activities [Figure 4].

## DISCUSSION

Multicentric giant cell tumor occurs more often than solitary lesions in young patients. Incidence of multicentric giant cell tumor is < 1%. Thirty cases are reported till 2006 of multicentric giant cell tumor.^2^ Out of them 11 cases (37%) presented with synchronous tumors. Six of these cases involved the knee (the distal part of the femur and proximal part of the tibia of the same limb.^2^ In one study, there were 10 lesions in the same person at presentation.^3^

But in our case, synchronous occurrence in all three bones - femur, tibia and patella around the knee was detected, which is a rare occurrence and hence requires reporting. In approximately 1% of cases, it manifests as multiple synchronous metachronus lesions in single or multiple bones.^4^–^6^

When the tumor is large and nearer to the subchondral bone plate, with intact articular cartilage, thorough curettage and filling the cavity with acrylic cement and tricortical bone graft will preserve cartilage, provide stability, allow early and full mobility.^7^

Sandwich technique is used to maintain smoothness of articular surface by introduction of gel foam; bone graft is used to support and prevent collapse of articular cartilage as the lesion is subchondral in nature. Cement is used to augment support by the bone graft to the articular surface. This together forms a sandwich at the lesion hence termed as Sandwich technique.

A thorough curettage with chemical cauterization with 5% phenol and 70% alcohol, followed by electric cauterization and cementing with polymethyl methacrylate cement destroys residual microscopic tumor cells, if any, by its chemical and exothermic reaction and hence reduces the chance of recurrence.^8^

## CONCLUSION

Multicentric giant cell tumor is a rare entity. Synchronous occurrence in all three bones around the knee still a rare occurrence hence reported. The case was managed by sandwich technique and resulted in good stability with excellent mobility at knee joint.
